# Meta-analysis of genome-wide association studies of hoarding symptoms in 27,651 individuals

**DOI:** 10.1038/s41398-022-02248-7

**Published:** 2022-11-15

**Authors:** Nora I. Strom, Dirk J. A. Smit, Talisa Silzer, Conrad Iyegbe, Christie L. Burton, René Pool, Mathieu Lemire, James J. Crowley, Jouke-Jan Hottenga, Volen Z. Ivanov, Henrik Larsson, Paul Lichtenstein, Patrik Magnusson, Christian Rück, Russell J. Schachar, Hei Man Wu, Sandra M. Meier, Jennifer Crosbie, Paul D. Arnold, Manuel Mattheisen, Dorret I. Boomsma, David Mataix-Cols, Danielle Cath

**Affiliations:** 1 Department of Psychology, Humboldt-Universität zu Berlin, Berlin, Germany; 2 Institute of Psychiatric Phenomics and Genomics (IPPG), University Hospital, LMU Munich, Munich, Germany; 3 Centre for Psychiatry Research, Department of Clinical Neuroscience, Karolinska Institutet & Stockholm Health Care Services, Region Stockholm, Sweden; 4 Department of Biomedicine, Aarhus University, Aarhus, Denmark; 5 Department of Psychiatry, Amsterdam University Medical Centers, Amsterdam, The Netherlands; 6 Amsterdam Neuroscience, Amsterdam, The Netherlands; 7 Department of Psychiatry, Hospital for Sick Children, Toronto, ON Canada; 8 Department of Psychosis Studies, Institute of Psychiatry, Psychology and Neuroscience, King’s College London, London, England; 9 Department of Genetics and Genomic Sciences, Icahn School of Medicine, Mount Sinai, New York, USA; 10 Department of Biological Psychology, Vrije Universiteit, Amsterdam, Netherlands; 11 Departments of Genetics and Psychiatry, University of North Carolina at Chapel Hill, Chapel Hill, USA; 12 Netherlands Twin Register, Biological Psychology, Vrije Universiteit, Amsterdam, The Netherlands; 13 Department of Medical Epidemiology and Biostatistics, Karolinska Institutet, Stockholm, Sweden; 14 School of Medical sciences, Örebro University, Örebro, Sweden; 15 Department of Psychiatry, Dalhousie University, Halifax, NS Canada; 16 Community Health & Epidemiology, Dalhousie University, NS Dalhousie, Canada; 17 The Mathison Centre for Mental Health Research & Education, Hotchkiss Brain Institute, Cumming School of Medicine, University of Calgary, Calgary, AB Canada; 18 Departments of Psychiatry and Medical Genetics, Cumming School of Medicine, University of Calgary, Calgary, AB Canada; 19 Amsterdam Public Health Research Institute, Amsterdam, The Netherlands; 20 Amsterdam Reproduction and Development Research Institute, Amsterdam, The Netherlands; 21 Rijksuniversiteit Groningen and Department of Psychiatry, University Medical Center Groningen, Groningen, Netherlands; 22 Department of specialized training, Drenthe Mental Health Care Institute, Assen, The Netherlands

**Keywords:** Genetics, Psychology

## Abstract

Hoarding Disorder (HD) is a mental disorder characterized by persistent difficulties discarding or parting with possessions, often resulting in cluttered living spaces, distress, and impairment. Its etiology is largely unknown, but twin studies suggest that it is moderately heritable. In this study, we pooled phenotypic and genomic data from seven international cohorts (*N* = 27,651 individuals) and conducted a genome wide association study (GWAS) meta-analysis of parent- or self-reported hoarding symptoms (HS). We followed up the results with gene-based and gene-set analyses, as well as leave-one-out HS polygenic risk score (PRS) analyses. To examine a possible genetic association between hoarding symptoms and other phenotypes we conducted cross-trait PRS analyses. Though we did not report any genome-wide significant SNPs, we report heritability estimates for the twin-cohorts between 26–48%, and a SNP-heritability of 11% for an unrelated sub-cohort. Cross-trait PRS analyses showed that the genetic risk for schizophrenia and autism spectrum disorder were significantly associated with hoarding symptoms. We also found suggestive evidence for an association with educational attainment. There were no significant associations with other phenotypes previously linked to HD, such as obsessive-compulsive disorder, depression, anxiety, or attention-deficit hyperactivity disorder. To conclude, we found that HS are heritable, confirming and extending previous twin studies but we had limited power to detect any genome-wide significant loci. Much larger samples will be needed to further extend these findings and reach a “gene discovery zone”. To move the field forward, future research should not only include genetic analyses of quantitative hoarding traits in larger samples, but also in samples of individuals meeting strict diagnostic criteria for HD, and more ethnically diverse samples.

## Introduction

Hoarding Disorder (HD) is one of the most recent mental disorders to be included in the DSM-5 [1] and ICD-11 [2]. Individuals with HD experience persistent difficulties parting with possessions, regardless of their value, due to a perceived need to save the items and distress associated with discarding them. This results in the accumulation of possessions that clutter active living areas and substantially compromise their intended use, causing clinically significant distress or impairment. Most people with HD also excessively acquire items that they do not need and experience distress if they are unable or are prevented from acquiring items (excessive acquisition specifier). Critically, these symptoms are not attributable to another medical or mental disorder, such as obsessive-compulsive disorder (OCD), psychosis or dementia [1, 2].

The prevalence of HD in the population is estimated to be approximately 1% to 2.5% for both men and women [3–6], but a much larger proportion of the population experience symptoms at various levels of severity, with estimates up to 6.7% [7] and 9% [8] in some studies. It is widely believed that the liability to hoarding symptoms (HS) is continuous in the general population, with clinically relevant HD at the extreme end of the spectrum [9].

HS typically appear in early-to-mid-adolescence and, in contrast to many other psychiatric disorders, symptom severity increases with age [10–13]. Psychiatric comorbidity is common in HD, with up to 70% of individuals having at least one additional disorder, most commonly anxiety and/or depression (DEP [14, 15]). Attentional problems are also common in individuals with HD [14, 16–18].

The etiology of HD is largely unknown, though likely to be multifactorial in nature and related to a complex interplay of genetic, neurobiological, and psychosocial factors. Family studies have consistently shown that HS run in families [15, 19–22]. Population-based twin studies have estimated the heritability of hoarding symptoms based on self-report questionnaires [3, 4, 7, 23–26]. In adults, heritability estimates range from 26 to 49%; the remaining variance was due to unique environmental factors and measurement error, whilst shared environmental factors appear to play a negligible role. In young people, large population-based samples of twins (*N* = 3974 twins, [3]; *N* = 25,523, [4]) reported higher heritability of hoarding symptoms amongst 15-year-old boys than in girls (33% vs 17%) and significant shared environment influences (22%) among female twins only, while Burton et al. [8] reported a heritability of 61% with no shared environment effect (221 twin pairs). Thus, it is possible that genetic and environmental influences on hoarding symptoms change across development, with shared environmental factors being more important in young people (particularly in girls).

Linkage and GWAS studies of HS have been rare thus far and have largely been conducted in small samples of OCD or Tourette syndrome patients. Candidate gene studies in individuals with OCD have suggested (largely non-replicated) associations between HS and a number of candidate variants [27–31]. Three previous modestly sized genome-wide linkage studies of HS in OCD or Tourette syndrome samples resulted in either no significant or conflicting results [21, 32, 33]. One study in OCD patients found linkage between HS and a region on chromosome 14q23-32 [21], and another linkage study in OCD patients found evidence for interaction with a region on chromosome 9q that houses *SLC1A1*, a glutamate transporter gene [32]. One GWAS of OCD symptom dimensions reported *SETD3*, a gene highly expressed in the brain and involved in apoptotic processes and transcriptomic changes, to be associated with HS [34]. Another GWAS focused on HS in a British twin cohort [35]. The sample included 3304 twins from the TwinsUK cohort, predominantly female (91.8%), with a mean age of 56.8 years. All participants completed the Hoarding Rating Scale-Self-Report (HRS-SR; [36]), a brief self-administered instrument consisting of five items (clutter, difficulty discarding, excessive acquisition, distress, and impairment). While no genome-wide significant loci were identified, two genomic loci on chromosomes 5 and 6 showed suggestive evidence for association with HS.

Larger samples are needed to increase power to detect significant genetic effects. Therefore, the current study aimed to conduct a GWAS meta-analysis of several large international cohorts from Sweden, the Netherlands, England, and Canada that included parent- or self-report hoarding scale data. We pooled data from seven population-based cohorts that together include 27,651 individuals (including 7012 twin-pairs), representing a more than eightfold increase in sample size compared to the previous study by Perroud et al. [35]. We followed up the results with gene-based and gene-set analyses, as well as leave-one-out hoarding symptom polygenic risk score (PRS) analyses and cross-trait PRS analyses to examine a possible genetic association between other phenotypes and HS.

## Materials and methods

### Cohorts and phenotype assessment

The Hoarding Symptom (HS) GWAS meta-analysis included individuals from seven different European-ancestry cohorts. Four cohorts are part of the Swedish Twin Registry (STR, [37]), namely different age groups of the *Child and Adolescent Twin Study in Sweden* (CATSS15, CATSS18, and CATSS24 [38]), and the *Young Adult Twins in Sweden Study* (YATSS [39]). CATSS is a prospective, longitudinal study of all twins born in Sweden since 1992. For CATSS, one measurement time point per individual was selected, preferring the measurement at age 24 over age 18 over age 15 if more than one measurement was completed. The other cohorts are from the Netherlands Twin Register (NTR, [40]), Spit for Science (SfS, [41, 42]), and TwinsUK (see Supplementary Material for detailed descriptions). Data from the TwinsUK cohort were included in a previous GWAS [35]. The cohorts are all population-based, predominantly including twins (except SfS), with a mean age-range between 11 and 57 (Table 1). Participants, or their parents, answered one of two questionnaires assessing HS.

**Table 1:** Overview of cohorts included in the GWAS meta-analysis of HS.

	STR-CATSS15	STR-CATSS18	STR-CATSS24	STR YATSS	NTR	SfS	TwinsUK
**N**	3605	3286	2313	2947	6839	5218	3443
N MZ twin pairs	241	256	191	552	866	–	317
N DZ twin pairs	1219	1137	685	348	425	–	775
N siblings	–	–	–	–	438	–	–
N parents	–	–	–	–	1361	–	–
% female	50%	56%	57%	63%	66%	48%	92%
Mean age ± SD	15.47 ± 0.36	18.56 ± 0.33	23.84 ± 0.32	23.93 ± 1.78	41.49 ± 15.19	10.92 ± 2.79	56.7 ± 12.6

For each individual cohort included in the HS meta-analysis (STR-CATSS15, STR-CATSS18, STR-CATSS24, STR-YATSS, NTR, SfS, TwinsUK), the table lists the total sample size included (N), the number of monozygotic twin pairs (N MZ twin pairs), the number of dizygotic twin pairs (N DZ twin pairs), the number of siblings (N siblings), the number of parents (N parents), the percentage of females and males in the total N (% females (males)), and the mean age with standard deviations (SD). Twins where only one twin participated were not counted as twins. NTR twin pairs include 10 multiplets, (31 individuals), 185 spouses of twin probands and 1153 twins without any other family member participating. Note that CATSS samples were later pooled across the three cohorts (CATSS15, CATSS18, CATSS24) for GWAS analysis, depending on the platform they were genotyped on (GSA, PsychChip).

In STR, NTR, and TwinsUK, HS were assessed using four (STR-YATSS, STR-CATSS24) to five items (STR-CATSS15, STR-CATSS18, NTR, TwinsUK) of the Hoarding Rating Scale Self-Report (HRS-SR; [43]), while in SfS parent- or self-reported hoarding traits were assessed using two items from the Toronto Obsessive Compulsive Scale (TOCS), a 21-item questionnaire described elsewhere [44, 45] (see Supplementary Table S1 for questionnaire details). Though the TOCS was originally designed to measure obsessive-compulsive symptoms, the two questions used here effectively reflect two core components of hoarding, namely acquisition of objects and difficulty discarding. To summarize across HRS-SR items, four items of the HRS-SR were used to calculate a one-factor model using a latent variable analysis with the R package lavaan [46] confirmatory-factor-analysis function. For STR item 4 of the HRS-SR was not assessed (“To what extent do you experience impairment in your life (daily routine, job/school, social activities, family activities, financial difficulties) because of clutter, difficulty discarding, or problems with buying or acquiring things?”), while for NTR and TwinsUK item 5 was not included (“To what extent do you experience emotional distress because of clutter, difficulty discarding or problems with buying or acquiring things?”). Fit indices of the one-factor model were compared to ensure that by dropping the respective item, still the same construct is measured for all cohorts. Standardized individual factor scores were calculated for the common factor model using the lavPredict function. Standardized individual factor scores were calculated for the common factor model using the lavPredict function. In case an individual was missing one item, the mean of the remaining three items was used to impute the missing value. If more than one item was missing, the individual was removed from the analysis. The two SfS Hoarding items of the TOCS were summed and standardized into a Z-score. To ensure reliable and valid symptom reporting, SfS participants <12 years of age with self-reported HS were excluded.

### Genome-wide association analysis

All participants were genotyped on SNP-arrays based on DNA from saliva or blood. One part of the STR-CATSS samples was genotyped on the PsychChip genotyping array (*N* = 8598), another part was genotyped on the GSA genotyping array (*N* = 606). For the GWAS analyses, the STR-CATSS cohorts (CATSS15, CATSS18, CATSS24) were pooled over each genotyping platform (GSA, PsychChip), forming two separate CATSS datasets (STR-CATSS-GSA and STR-CTASS-PC). Each of the six datasets (STR-CATSS-GSA, STR-CATSS-PC, STR-YATSS, NTR, SfS, and TwinsUK) underwent stringent quality-control (QC), including the removal of non-European ancestry outliers based on PCA and imputation using the HRC [47] (STR, NTR) or the 1000 G [48] (SfS, TwinsUK) reference sets (see Supplementary Material for more details). After genotyping, quality control, and imputation of each cohort, STR included 12,151, NTR 6839, SfS 5218, and TwinsUK 3443 (total *N* = 27,651) individuals with complete genotypic and phenotypic information.

A linear mixed modeling GWAS was conducted within each cohort using GCTA-fastGWA [49, 50]. For STR, NTR, and TwinsUK a sparse Genetic Relatedness Matrix (GRM) was calculated and the first 10 principal components, sex, age, age squared, and genotyping batches were used as covariates. In a sparse GRM all off-diagonal values below 0.05 are set to 0, thereby accounting for the close relatedness of individuals in the data. For SfS, analyses were performed on unrelated individuals; the first enrolled sibling from each family was selected for further analysis. The analysis was performed with a full GRM and sex, age, respondent (parent vs. child reporting), genotyping array, principal components 1–3 and projected principal components 1–3 (see Supplement for details) as covariates.

Next, the resulting GWAS summary statistics were cleaned and harmonized. All variants were filtered on minor allele frequency (MAF) > 1%, and imputation-quality score > 0.8. All datasets were aligned to the HRC-reference. In case alleles were reported on different strands, they were flipped to the orientation in the HRC reference. Strand ambiguous A/T and C/G SNPs were removed if their MAF was ≥0.4. Remaining ambiguous SNPs were strand aligned by comparing MAF to the HRC reference [47]. We then used METAL [51] within the Rapid Imputation for COnsortias PIpeLIne (Ricopili) [52] to conduct an inverse variance weighted meta-analysis. The genomic control factor (Lambda and Lambda1000) was inspected for each individual cohort to detect any residual population stratification or systematic technical artifacts. Also, the linkage disequilibrium (LD) score regression (LDSC) [53] intercept was inspected as an alternative measure of test statistic inflation. The genome-wide significance threshold was set at 5 × 10^–8^.

### Heritability

Heritability estimates of each individual cohort were extracted from the GCTA association output. GCTA uses a restricted maximum likelihood (REML) approach [54] to estimate heritability in the GRM that is supplied to correct for relatedness in the linear association test. This means that for the twin cohorts (STR, NTR, and TwinsUK), the heritability was based on the sparse GRM, while for the unrelated SfS cohort, heritability was based on the full GRM. For all heritability estimates, the same covariates as in the GWAS analyses were used. We further used LDSC [53] to calculate the SNP heritability of the HS GWAS meta-analysis. The SNP heritability in LDSC is based on the estimated slope from the regression of the SNP effect sizes from the GWAS on the LD score.

### Gene-based and gene-set analyses

We carried out a Multi-marker Analysis of GenoMic Annotation (MAGMA) [55] v1.08 as implemented in the web-based tool Functional Mapping and Annotation of Genome-Wide Association Studies (FUMA) [56] v1.3.7 to test genetic associations at the gene level for the combined effect of SNPs in or near protein coding genes. Gene-based *p*-values were computed by mapping SNPs to their corresponding gene(s) based on their position in the genome. Positional mapping was based on ANNOVAR annotations and the maximum distance between SNPs and genes was set to the default setting of 10 kb. Based on the results of gene analysis, competitive gene-set analysis was performed with default parameters. The 15,496 gene-sets were obtained from MsigDB v7.0, including ‘*Curated gene sets*’ consisting of nine data resources including KEGG, Reactome, and BioCarta, and *GO terms* consisting of three categories (biological processes, cellular components, and molecular functions).

### Cross-trait polygenic risk score (PRS) analyses

To explore the genetic relationship between HS and other phenotypes, we calculated a range of PRSs based on large-scale GWAS summary statistics. We selected mainly studies of psychiatric disorders, i.e., OCD [57], DEP [58], schizophrenia (SCZ) [59], autism spectrum-disorder (ASD) [60], attention-deficit-hyperactivity disorder (ADHD) [61], and educational attainment (EA) [62]. PRS were computed in PRSice2 [63] for each cohort. The PRS scores were calculated as the weighted sum of the risk allele dosages at pre-selected *p*-value thresholds based on the reported thresholds in the primary publications (EA: *P* = 1; ADHD, ASD, OCD, SCZ: *P* = 0.01; DEP: *P* = 0.5). For STR, PRS analyses were conducted separately for the three datasets (STR-CATSS-GSA, STR-CATSS-PC, STR-YATSS) and were subsequently merged.

To evaluate the relationship between each PRS score and HS in every cohort, we employed generalized estimating equations (GEE) in R (STR, NTR, and TwinsUK). The GEE analysis accounts for the relatedness in the datasets. As SfS did not contain any related individuals, we carried out linear regression, as implemented within the PRSice2 pipeline. Again, the same covariates that were previously used in the respective GWASs were included.

PRS estimates per discovery phenotype were summarized across all target datasets by means of an inverse variance meta-analysis using the metagen package in R. We examined heterogeneity in PRS estimates across the cohorts with Cochran’s *Q* test [64] and Higgin’s *I*² [65, 66]. Q is calculated as the weighted sum of the squared differences between individual cohort effects and the pooled effect across cohorts, with the weights being those used in the pooling method. The *I*² statistic describes the percentage of variation across studies that is due to heterogeneity rather than sample variation and does, unlike Q, not inherently depend upon the number of measures included in the meta-analysis. Regardless of observed heterogeneity, we calculated a fixed effects model to evaluate the association of each PRS with HS. If there was considerable observed heterogeneity across study sites (*I*² > 0.5 and/or *P*_Q_ < 0.05), we further calculated a random effects model.

### Compatibility of cohorts

To identify if the summary statistics from any of the included cohorts substantially deviated from the others, we performed leave-one-out (LOO) GWAS meta-analyses and carried out a set of sensitivity analyses. With the replication module of the Ricopili pipeline, sign tests on the top SNPs (inclusion threshold of *p* = 0.0001, *p* = 0.00001, and *p* = 0.000001) were performed between each pair-wise combination of cohorts as well as between LOO meta-analyses and the left-out cohort to identify any cohort in which the GWAS results markedly deviated from the rest of the cohorts. Sign-tests allow for the quantification of independent genomic regions that have the same direction of effect in two separate summary statistics. The output, in form of a ratio, gives an estimate of the percentage of genomic regions with the same direction of effect in the two compared datasets. A sign-test is a binomial test with the null-hypothesis = 0.5, with a ratio above 0.5 indicating convergence, and a ratio below 0.5 indicating divergence. While certain fluctuations in the sign-tests across different *p*-value thresholds are expected, depending on the true association of each SNP with the phenotype, we mainly aimed to assess whether a specific cohort or age-group markedly deviated from the rest.

To evaluate the relationship between the PRS scores of each LOO GWAS and standardized HS scores in the left-out cohort, we conducted LOO PRS analyses, following the same procedure as for the cross-trait PRS analyses described above (see previous method-section on cross-trait PRS analyses for details).

## Results

### Phenotype normalization

The distributions of each cohorts’ sum-scores have been maximally normalized (distribution of the item- and total raw hoarding scores are shown in Supplementary Figs. S1–S6, distribution of the one-factor model scores (STR, NTR, and TwinsUK) and standardized scores (SfS) are shown in Supplementary Figs. S7–S10). An over-representation of zero sum-scores indicates censoring. The model-fit of the one-factor models in the latent variable analysis of the HRS-SR for NTR (CFI = 0.999, SRMR = 0.014) and STR (STR-GSA: CFI = 0.995, SRMR = 0.032; STR-PC: CFI = 0.997, SRMR = 0.020; STR-YATSS: CFI = 0.997, SRMR = 0.020) were excellent, thereby demonstrating that all items of the HRS-SR highly load onto one common factor and psychometrically measure the same construct, allowing us to drop one item of the HRS-SR per analysis. The two TOCS hoarding items in the SfS data could not be assessed for model fit, however, the two items were highly comparable in wording to items 2 and 3 of the HRS.

### Genome-wide association results

**Fig. 1:**
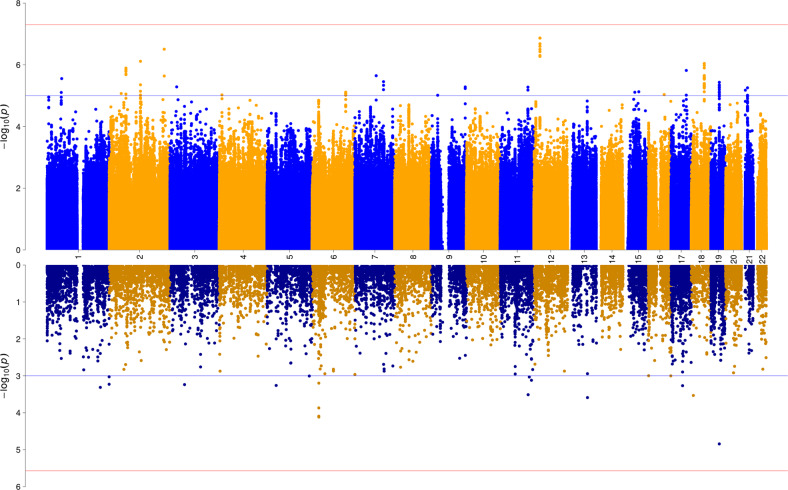
Miami plot of the association results from the GWAS meta-analysis (upper panel) and of the gene-wide association analysis (lower panel) of HS. The *y*-axes represent −log10 *P* values for the association of SNPs/genes with HS. The *x*-axis represents chromosomes 1 to 22. In the upper plot, the *P*-value threshold for genome-wide significance (5 × 10^–08^) is represented by the horizontal red line, suggestive significance (*p* = 1 × 10^–05^) by the blue line. In the lower panel, Bonferroni-corrected gene-wide significance (*p* = 2.682 × 10^–06^) is represented by the horizontal red line, suggestive gene-wide significance (*p* = 1 × 10^–03^) is indicated by the blue horizontal line.

The final dataset included 27,651 individuals with complete phenotypic and genotypic data and 6,541,342 autosomal SNPs. No significant inflation was observed (*λ* = 1.024, *λ*_1000_ = 1.001, LDSC intercept = 1.0173, see Supplementary Fig. S11 for QQ plot). No SNP exceeded the genome-wide significance threshold (see Fig. 1 for a Miami-plot including the Manhattan-plot of the GWAS in the upper panel). The SNPs with the lowest *p*-values (<1 × 10^–6^) were rs117321479 (*P* = 1.36 × 10^–7^) on chromosome 12, rs78426839 (*P* = 3.12 × 10^–7^) and rs7567224 (*P* = 7.70 × 10^–7^) on chromosome 2, and rs72927972 (*P* = 9.09 × 10^–7^) on chromosome 18 (see Supplementary Figs. S12–S15 for regional association plots and forest plots). The region tagged by rs117321479 spans 57.6 kb (LD *r*² > 0.6) and entails the gene *SOX5*. The region tagged by rs78426839 spans 22.1 kb (LD *r*² > 0.6) and entails the genes *TUBA4B*, *DNAJB2*, *PTPRN*, *MIR153-1*, *RESP18*, and *DNPEP*. The region tagged by rs7567224 spans 24.40 kb (LD *r*² > 0.6) and entails the gene *CNTNAP5*, while the region tagged by rs78426839 spans 94.3 kb (LD *r*² > 0.6). In addition, 19 independent SNPs with *p* < 1 × 10^–5^ were identified (see Supplementary Table S2 for a list of corresponding association results).

### Heritability

For the twin cohorts, the additive genetic variance of HS, estimated based on the sparse genetic relatedness matrices, ranged between 0.26 (NTR) and 0.48 (TwinsUK), with estimates for the STR cohorts in between (STR-CATSS15: 0.47, STR-CATSS18: 0.29, STR-CATSS24: 0.35, STR-YATSS = 0.28). Note that the SNP-heritability estimates based on the twin cohorts are largely driven by the twin resemblance (~0.5 between DZ twins and siblings, 1.0 for MZ twins, and 0 between unrelated individuals). The heritability for SfS, only including unrelated individuals, was 0.11 (SE = 0.057, *P* = 0.0303). The SNP-based heritability estimate of the GWAS meta-analysis using LDSC resulted in a total observed-scale heritability of 0.019 (SE = 0.016, *Z* = 1.18, *P* = 0.235).

### Gene-based analyses

We conducted gene-based tests to determine whether any protein-coding gene carries a load of common variation associated with HS. SNPs were mapped to 18,646 protein coding genes obtained from Ensembl build 85. No gene reached the Bonferroni-corrected significance threshold of *p* = 0.05/18,646 = 2.682 × 10^–6^ (see Fig. 1 for a Miami-plot including the Manhattan-plot of the gene-based test in the lower panel, and Supplementary Fig. S11 for a QQ-plot). Also, none of 15,483 tested gene-sets reached the Bonferroni-corrected significance threshold.

### Cross-trait PRS analyses

To evaluate the genetic overlap between HS and other potentially related phenotypes of HS, we conducted PRS analyses. Publicly available summary statistics of OCD, DEP, SCZ, ASD, ADHD, and EA served as discovery datasets, with each HS cohort as the target dataset. GEE (STR, NTR, TwinsUK) and linear regression analysis (SfS) revealed Bonferroni-corrected significant (*p* < 0.05/7 = 0.00714) associations between HS and PRSs based on all DEP, SCZ and EA, though not consistently across all target cohorts (see Supplementary Table S3). In a meta-analysis summarizing PRS results across all HS cohorts, the PRS for SCZ showed a significant association with HS (*P*_fixed_ = 2.43 × 10^–06^, *P*_random_ = 0.00422). When not taking the heterogeneity of the individual PRS estimates into account, the meta-analyzed PRSs of ASD and EA also showed significant associations with HS (ASD: *P*_fixed_ = 0.00426; EA: *P*_fixed_ = 1.15E^−05^). As the associations with the PRS of EA showed substantial (*I*² = 0.76598, *P*_Q_ = 0.00504) heterogeneity, we further conducted random effects models to examine if the association with HS remains significant when accounting for this heterogeneity, resulting in a nominally significant association (EA: *P*_random_ = 0.00765). The associations with the PRS of DEP, OCD, and ADHD did not show any significant associations with HS in the meta-analysis (see Table 2 for all results).

**Table 2:** Depicted are results from the PRS meta-analysis for HS (leave-one-out), obsessive-compulsive disorder (OCD), depressive disorder (DEP), schizophrenia (SCZ), autism-spectrum disorder (ASD), attention-deficit hyperactivity disorder (ADHD), and educational attainment (EA) for pre-selected *p*-value thresholds (*P*_threshold_), meta-analyzed across all target datasets (STR, NTR, SfS, TwinsUK).

Discovery	P_threshold_	N_effective_	Heterogeneity		*P*_fixed_	*P*_random_
			*I*²	*P*_Q_		
**Hoarding Symptoms (Leave-one-out)**						
HS	0.5	25,389	0	0.925	0.553	
**Cross-trait**						
OCD	0.1	25,465	0	0.629	0.674	
DEP	0.05	25,606	0.827	0.001	0.015	0.586
SCZ	0.1	25,547	0.450	0.142	**2.43E**^**−06**^	
ASD	0.1	25,977	0.267	0.252	**0.004**	
ADHD	0.1	25,938	0.555	0.081	0.213	0.816
EA	1	25,307	0.766	0.005	**1.15E**^**−05**^	0.008

As measures of heterogeneity of PRS associations across all target datasets, Higgin’s *I*² statistic and the *p*-value for Cochran’s *Q* test (*P*_Q_) are reported. *P*_fixed_ and *P*_random_ list the *p*-values of a fixed and a random-effects model, respectively. A random effects model was only calculated if there was substantial (*I*² > 0.5 and/or *P*_Q_ < 0.05) heterogeneity across the datasets. The effective sample size (*N*_effective_) is summed over the effective sample size of every target dataset. For STR, NTR, and TwinsUK the effective *N* was determined based on the actual N (including family members) weighted by the ratio of the squared SE from the GEE sandwich-corrected model and the naive model (no correction), for SfS the total sample *N* was used. Bonferroni-corrected significant *p*-values (<0.05/7 = 0.00714) are in bold.

### Compatibility of cohorts

No genome-wide significant heterogeneity was observed in the HS GWAS meta-analysis (see Supplementary Fig. S16 for Manhattan-plot and QQplot of the heterogeneity test). A range of sensitivity analyses, including LOO GWAS analyses (see Supplementary Figs. S17–S20 for LOO Manhattan plots and QQ-plots), and subsequent sign-test analyses and LOO PRS analyses provided further evidence that there was no systematic and substantial heterogeneity across the different cohorts.

First, we conducted sign tests at three different *p*-value thresholds (1 × 10^–06^, 1 × 10^–05^, 1 × 10^–04^) between each pair-wise combination of STR, NTR, SfS, and TwinsUK (see Supplementary Table S4). Second, to determine if there was any pronounced age-related effect in the STR or NTR data, we conducted sign-test analyses between LOO GWAS analyses of age-separated STR sub-cohorts (Supplementary Table S5), age-separated NTR sub-cohorts (Supplementary Table S6) and the respective left-out cohort. The STR cohorts were divided into four age groups, pertaining to their division into separate phenotyping rounds (CATSS15 mean age = 15.46; CATSS18 mean age = 18.56; CATSS24 mean age = 23.84; YATSS mean age = 23.93), while the NTR data was separated into three age groups (group1 < 30 years; group2 30–45 years; group3 > 45 years). The sign test results at the *p*-value threshold of *p* = 1 × 10^–06^ identified very few independent genomic regions (0–6) and are therefore difficult to interpret. While the ratios of the sign tests for the other two *p*-value thresholds (*p* = 1 × 10^–05^ and *p* = 1 × 10^–04^) varied between 0.2 and 0.8, there is no apparent pattern indicating a systematic deviation of one cohort from the rest.

The LOO PRS analyses did not show a significant association between any of the PRSs and the HS score of the left-out cohort. While this could suggest heterogeneity across the cohorts, it is likely indicating a low power for this analysis considering that each discovery cohort had a rather low sample size and we only saw significant associations with PRS based on GWASs with considerably higher sample sizes (SCZ, DEP, EA). See Supplementary Tables S3 and 2 for results.

## Discussion

With 27,651 included individuals we conducted the largest GWAS study of HS in the population to date. Although we could not report any genome-wide significant SNPs, we found a significant contribution of common genetic factors to HS as indicated by a substantial genetic SNP heritability of 11% (*P* = 0.0303) in one of our cohorts (SfS) with unrelated samples. It suggests that, with sufficient power, specific genetic variants that are associated with HS will be eventually identified. SNP-based heritability of the meta-analysis as calculated with LDSC was low (*h*² = 0.019, SE = 0.016, *Z* = 1.18) and non-significant. We therefore did not conduct genetic correlation analyses as it is suggested to have a heritability Z-score of above 1.5 (optimal > 4) in order for the analysis to be meaningful [53, 67]. We also found a significant genetic variance component in the twin family cohorts, ranging from 28 to 48%. These heritability estimates may be largely driven by the relatedness between the samples and are indeed more comparable to pedigree-based twin-heritability estimates. Ivanov et al. [4] reported twin-heritability estimates of 41%, 31%, and 29% for the Swedish CATSS15, CATSS18, and YATSS cohorts, respectively, while in this study we report heritability estimates of 47%, 29%, and 28% for the same cohorts, respectively.

The region tagged by the SNP with the lowest *p*-value in our analysis (rs117321479) entails the gene *SOX5*, which is a member of the *SOX* family of transcription factors involved in the regulation of embryonic development and in the determination of cell fate [68]. *SOX5* has been identified as a gene with a high pleiotropic effect on a broad spectrum of psychiatric disorders and has been associated especially with ASD, BIP, MD, and SCZ, and to a lesser degree also with OCD, TS, ADHD, and AN [69]. Overall, *SOX5* has been genome-wide significantly associated with 64 phenotypes, spanning a wide range of domains including psychiatric (e.g. neuroticism [70, 71]), skeletal (e.g. height [72]), reproductive (e.g. age at menarche [73]), metabolic (e.g. hip circumference, BMI [70]), and environmental traits (e.g. household income [70], educational attainment [62]). Given this possible pleiotropy, it suggests that *SOX5* might be involved in susceptibility to general psychopathology.

By current standards [58–61], the size of our GWAS was modest and no significant effects were observed in the leave-one-sample-out analysis of PRS associated with HS, indicating that the lack of finding genome-wide hits is most likely attributable to a lack of power. We further observed a rather high number of individuals (28.8%) with zero scores on the hoarding rating scales, leading to a relatively low variance of symptom scores in our dataset, and our datasets included a high number of twins, both of which likely reduced the effective sample size and contributed to the power issue.

Another reason why we did not find a significant signal may lie in differences between the included study cohorts, beyond any heterogeneity that we were able to detect. Possible sources of heterogeneity across the datasets include age, instruments used to assess HS, or ascertainment of data. The SfS cohort stands out compared to the other cohorts with regards to age and questionnaire used. We addressed this issue by applying a framework of sensitivity and heterogeneity analyses. Neither in the genome-wide heterogeneity test, nor in the sign-tests or LOO PRS analyses did we observe a pattern that indicated a systematic deviation of one cohort from the rest. We therefore concluded that a meta-analysis of the cohorts is warranted and that the lack of signal is indeed most likely attributable to a lack of power. As most of the individuals included in the cohorts in this study were relatively young compared to the age when the full disorder debuts, there is the possibility that HS reported by younger individuals are the outcome of a somewhat different phenotype than HS reported at an older age.

Cross-trait PRS analyses showed significant results. The genetic risk for schizophrenia was significantly associated with HS, while we found suggestive evidence for an association with autism spectrum disorder and educational attainment. This suggests that a well-powered GWAS such as those used in the cross-trait PRS analyses (SCZ: *N*_effective_ = 214,576; ASD: *N*_effective_ = 44,367; EA: *N*_effective_ = 245,621) can pick up genetic signals that may be associated with hoarding symptoms. We consider this a further indicator that additional power is needed to obtain reliable signal in a quantitative trait GWAS for HS. The lack of significant associations with the most described comorbidities in HD (OCD, depression, anxiety, ADHD) was unexpected and possibly attributable to the currently modest size of the discovery samples.

Previous twin studies of HS have shown a clear role of genetic factors [3, 4, 7, 23–26]. The current study confirmed these observations with a significant SNP-based heritability of HS. It is not entirely surprising that we did not discover any genome-wide significant associations, as other studies with similar sample sizes that investigated quantitative measures of symptoms also lacked power to discover associations [74, 75]. Nevertheless, the quantitative ADHD symptom GWAS by Middeldorp et al. [75] showed a high genetic correlation and strong concordance at individual loci with clinical ADHD [61], thereby supporting the hypothesis that clinically diagnosed cases are the extreme of a quantitative symptom trait and further demonstrating the usefulness and importance of quantitative assessment of symptoms in the population.

A further limitation is that hoarding symptom scales may reflect heterogeneous disorders. It is known that many different psychiatric disorders can cause hoarding-like symptoms, such as schizophrenia, OCD, or severe depression. HD is essentially a diagnosis of exclusion (DSM-5, ICD-11). As in any study based on self-administered instruments, it was not possible to rule out these other causes of HS. It will further be of interest to determine the extent to which HS in the population and clinical HD share the same genetic susceptibility. However, no case-control GWAS of HD exists, nor is HD assessed in large datasets like UK BioBank [76, 77], which is unfortunate given the relatively high prevalence of around 2.5% for HD [6] and high individual and societal cost [78, 79]. Thus, for the time being, the study of HS in the population may be the only feasible approach to understanding the genetics of HD.

To conclude, we found that HS are heritable, confirming and extending previous twin studies. Nevertheless, we had limited power to detect any genome-wide significant loci. Much larger samples will be needed to further extend our findings and reach a “gene discovery zone”. Further, additional samples should be more ethnically diverse to ensure that results are relevant to individuals of non-European ancestry [80]. Future research should include the collection of DNA samples from individuals with HS, as well as samples from strictly diagnosed HD patients.

## Supplementary information

Supplementary Information

Supplementary Tables

## Data Availability

The meta-analyzed summary statistics will be made available via the Psychiatric Genomics Consortium Download page (https://www.med.unc.edu/pgc/download-results/).
