# Correction: Meta-analysis of genome-wide association studies of hoarding symptoms in 27,537 individuals

**DOI:** 10.1038/s41398-022-02288-z

**Published:** 2022-12-22

**Authors:** Nora I. Strom, Dirk J. A. Smit, Talisa Silzer, Conrad Iyegbe, Christie L. Burton, René Pool, Mathieu Lemire, James J. Crowley, Jouke-Jan Hottenga, Volen Z. Ivanov, Henrik Larsson, Paul Lichtenstein, Patrik Magnusson, Christian Rück, Russell J. Schachar, Hei Man Wu, Sandra M. Meier, Jennifer Crosbie, Paul D. Arnold, Manuel Mattheisen, Dorret I. Boomsma, David Mataix-Cols, Danielle Cath

**Affiliations:** 1 Department of Psychology, Humboldt-Universität zu Berlin, Berlin, Germany; 2 Institute of Psychiatric Phenomics and Genomics (IPPG), University Hospital, LMU Munich, Munich, Germany; 3 Centre for Psychiatry Research, Department of Clinical Neuroscience, Karolinska Institutet & Stockholm Health Care Services, Region Stockholm, Sweden; 4 Department of Biomedicine, Aarhus University, Aarhus, Denmark; 5 Department of Psychiatry, Amsterdam University Medical Centers, Amsterdam, The Netherlands; 6 Amsterdam Neuroscience, Amsterdam, The Netherlands; 7 Department of Psychiatry, Hospital for Sick Children, Toronto, ON Canada; 8 Department of Psychosis Studies, Institute of Psychiatry, Psychology and Neuroscience, King’s College London, London, England; 9 Department of Genetics and Genomic Sciences, Icahn School of Medicine, Mount Sinai, New York, USA; 10 Department of Biological Psychology, Vrije Universiteit, Amsterdam, Netherlands; 11 Departments of Genetics and Psychiatry, University of North Carolina at Chapel Hill, Chapel Hill, USA; 12 Netherlands Twin Register, Biological Psychology, Vrije Universiteit, Amsterdam, The Netherlands; 13 Department of Medical Epidemiology and Biostatistics, Karolinska Institutet, Stockholm, Sweden; 14 School of Medical sciences, Örebro University, Örebro, Sweden; 15 Department of Psychiatry, Dalhousie University, Halifax, NS Canada; 16 Community Health & Epidemiology, Dalhousie University, NS Dalhousie, Canada; 17 The Mathison Centre for Mental Health Research & Education, Hotchkiss Brain Institute, Cumming School of Medicine, University of Calgary, Calgary, AB Canada; 18 Departments of Psychiatry and Medical Genetics, Cumming School of Medicine, University of Calgary, Calgary, AB Canada; 19 Amsterdam Public Health Research Institute, Amsterdam, The Netherlands; 20 Amsterdam Reproduction and Development Research Institute, Amsterdam, The Netherlands; 21 Rijksuniversiteit Groningen and Department of Psychiatry, University Medical Center Groningen, Groningen, Netherlands; 22 Department of specialized training, Drenthe Mental Health Care Institute, Assen, The Netherlands

**Keywords:** Genetics, Psychology

Correction to: *Translational Psychiatry* 10.1038/s41398-022-02248-7, published online 15 November 2022

The original version of this article contained a typo in the text. The correct over-all sample size is 27651 instead of 27537 (with one included cohort, NTR, including *N* = 6839 individuals instead of the reported *N* = 6725). All analyses, including the main GWAS and all secondary analyses, were conducted with the correct sample size, this is simply a reporting error. The original article has been corrected.

