# Supplementary material for: Meta-analysis of genome-wide association studies of hoarding symptoms in 27,651 individuals
Source: Transl Psychiatry. 2022 Nov 15;12:479. doi: 10.1038/s41398-022-02248-7 (PMC9666541; doi:10.1038/s41398-022-02248-7)
Supplement: Supplementary file 2 — Supplementary Tables [file 41398_2022_2248_MOESM2_ESM.pdf]

Supplementary Table S1: Overview of Hoarding Rating Scale Self-Report (HRS-SR) questions in STR, NTR, and TwinsUK and the hoarding items of the Toronto Obsessive Compulsive Scale (TOCS) used in SfS.

	**STR**	**NTR**	**TwinsUK**	**SfS**
**Questionnaire**	HRS-SR	HRS-SR	HRS-SR	TOCS
**Language**	Swedish	Dutch	English	English
**Responder**	Self-reported	Self-reported	Self-reported	Self-reported, parent-reported
**Time Period**			Retrospective over previous year	
**Possible range of answers**	1-5	0-8	0-8	
**item 1**	To what extent do you have difficulty discarding (or recycling, selling, giving away) ordinary things that other people would get rid of? (Original: Hur svårt har du att slänga vardagliga saker som andra lätt gör sig av med?)	Because of the clutter or number of possessions, how difficult is it for you to use the rooms in your home?	In the last year… Because of the clutter or number of possessions, how difficult is it for you to use the rooms in your home?	Collect useless objects
**item 2**	To what extent do you currently have a problem with collecting free things or buying more things than you need or can use or can afford? (Original: Hur stora problem har du med att du samlar på saker som är gratis eller att du köper mer saker än du behöver eller har råd med?)	To what extent do you have difficulty discarding (or recycling, selling, giving away) ordinary things that other people would get rid of?	In the last year…To what extent do you have difficulty discarding (or recycling, selling, giving away) ordinary things that other people would get rid of?	Have difficulty throwing things away
**item 3**	Because of the clutter or number of possessions, how difficult is it for you to use the rooms in your home? (Original: Hur svårt är det för dig att använda ditt rum och/eller dina utrymmen på grund av röran eller mängden saker?)	To what extent do you currently have a problem with collecting free things or buying more things than you need or can use or can afford?	In the last year…To what extent do you currently have a problem with collecting free things or buying more things than you need or can use or can afford?	-
**item 4**	To what extent do you experience emotional distress because of clutter, difficulty discarding or problems with buying or acquiring things? (Original: Hur känslomässigt upprörd blir du för att det är rörigt, eller dina svårigheter med att slänga, köpa eller skaffa saker?)	To what extent do you feel impaired (for example in your daily routine, job/eductaion, social activities) because of clutter, difficulty throwing things away, or problems with buying or acquiring things?	In the last year…To what extent do you experience impairment in your life (daily routine, job/school, social activities, family activities, fmancial difficulties) because of clutter, difficulty discarding, or problems with buying or acquiring things?	-

Supplementary Table S2: LD-independent genomic regions with p < 1x10-5 in the HS meta-analysis and their associated genes. Listing for each single-nucleotide polymorphism (SNP) the respective chromosome (CHR), hg19 basepair-position (BP), association p-value (P), beta value from the association for allele 1 (Beta), standard error of the Beta (SE), effect allele and reference allele (A1/A2), frequency of allele 1 (FRQ), imutation quality score (INFO), number of individuals per SNP (N), direction of effect for each study (in the order STR-CatssGSA, STR-CatssPC, SfS, TwinsUK, NTR, STR-Yatss), a list of all variants with LD-r2 > 0.1 to the index SNP (friends(.1).p0.001), in brackets LD-r2 and distance in kb sorted by LD-r²; left and right margin of region (defined by LD friends) (range.left, range.right); span(kb)=right margin - left margin (in kb); same as before but with LD-r2 of 0.6 (friends(.6).p0.001, range.left.6, range.right.6, and span.6(kb)); list of entries in NHGRI GWAS catalogue among entries in column friends(.6) (gwas_catalog_span.6); and list of genes within the region of friends.6 (±50 kb), in brackets distance to index SNP in kb (genes.6.50kb(dist2index)).

SNP	CHR	BP	P	Beta	SE	A1A2	FRQ	INFO	N	Dir	LD-friends(0.1).p0.001	range.left	range.right	span(kb)	LD-friends(0.6).	range.left.6	range.right.6	span.6(kb)	gwas_catalog_span.6	genes.6.50kb(dist2index)	N.genes.6.50kb
rs117321479	12	23775967	1.36E-07	0.127901	0.0243	G/A	0.0277	0.987	20,812	----?-	rs117855589(0.998/-0.218),rs117847767(0.995/-1.44),rs41488244(0.981/-3.37),rs117715560(0.98/-7.83),rs118067810(0.979/-1.58),rs41349746(0.978/-6.48),rs80331093(0.944/4.85),rs80241699(0.792/-14.9),rs9971956(0.713/10.1),rs73279911(0.711/15.1),rs116968442(0.709/28.1),rs1002815(0.709/11.4),rs12297318(0.709/23.9),rs11047024(0.708/28.1),rs11047019(0.708/21.5),rs12306256(0.707/26.7),rs11047018(0.706/21.4),rs12319311(0.705/33.1),rs11047029(0.705/34),rs11047025(0.705/29.3),rs16926496(0.704/15.7),rs74071391(0.704/18.5),rs11047034(0.668/42.6)	23761067	23818567	57.5	rs117855589(0.9	23761067	23818567	57.5	-	SOX5(0.0)	1
rs78426839	2	220170598	3.12E-07	0.1162	0.0227	A/G	0.0378	0.91	15,594	++?+?+	rs34561714(0.806/22.1)	220170598	220192698	22.1	rs34561714(0.80	220170598	220192698	22.1	/(0.806)rs34561714;Heigl	TUBA4B(0.0),DNAJB2(0.0),PT	6
rs7567224	2	125107948	7.70E-07	-0.0349021	0.0071	C/T	0.411	0.994	27,651	++++++	rs56405601(0.742/-3.01),rs2420840(0.718/21.4),rs62171019(0.71/5.04),rs72839455(0.619/-3.09),rs7582767(0.587/0.416),rs7609391(0.587/0.504),rs59503656(0.58/5.74),rs7581635(0.579/12.6),rs62171016(0.514/2.33),rs4848240(0.496/25.3),rs11123037(0.365/23.1),rs10496629(0.327/26.9),rs7578650(0.301/5.03),rs779999(0.292/0.077),rs780045(0.289/-5.38),rs780048(0.289/-13.8),rs780046(0.289/-6.59),rs780000(0.288/-2.31),rs796641(0.279/0.243),rs811902(0.268/-4.72),rs780057(0.266/23.5),rs720477(0.266/19.6),rs780059(0.265/25.6),rs779981(0.265/18),rs780060(0.262/27.4)	125094148	125135348	41.2	rs56405601(0.74	125104858	125129348	24.49	-	CNTNAP5(0.0)	1
rs72927972	18	51528445	9.09E-07	0.0461963	0.0094	A/C	0.165	0.993	27,651	-+++++	rs72927946(0.997/-4.14),rs72926096(0.973/-11.6),rs72927915(0.973/-8.7),rs72926051(0.965/-18.4),rs72924148(0.963/-35.3),rs1498146(0.963/-43),rs72922092(0.962/-44.1),rs11660673(0.962/-41.6),rs142123239(0.957/-22.3),rs74652159(0.956/-62.1),rs72920351(0.956/-62.4),rs72920352(0.956/-62.3),rs72920339(0.956/-63.3),rs72929977(0.952/13.6),rs11659750(0.937/-6.26),rs72931916(0.937/21.6),rs79689252(0.893/-21),rs11661683(0.89/-23.4),rs75520509(0.88/-61.9),rs11659777(0.869/-72.7),rs17536164(0.658/-7.26),rs9304456(0.655/-7.82),rs28451198(0.654/-8.2),rs4502292(0.646/-16.1),rs28630974(0.644/-8.57),rs9955392(0.643/-12.2),rs16957645(0.643/-11.4),rs9955809(0.643/-11.6),rs4260126(0.643/-15.8),rs9962069(0.643/-10.1),rs7242057(0.643/-15.9),rs2133095(0.643/-13.7),rs76996980(0.643/-11.5),rs7236457(0.643/-10.5),rs7235741(0.643/-10.2),rs9946008(0.643/-14.6),rs4453581(0.643/-9.41),rs9951640(0.643/-16.8),rs7241181(0.643/-9.23),rs8092211(0.643/-16.9),rs34901938(0.643/-15.6),rs7231673(0.643/-10.6),rs16957651(0.643/-10.9),rs7239505(0.643/-13.4),rs16957628(0.643/-15.5),rs9947376(0.643/-11.5),rs7236245(0.643/-10.1),rs9955618(0.643/-11.7),rs9944731(0.643/-11.9),rs8091906(0.643/-17),rs7241585(0.643/-16.2),rs9958112(0.643/-11.5),rs16957626(0.643/-15.5),rs11664112(0.641/-17.1),rs72922067(0.641/-47.3),rs1039187(0.641/-51.1),rs11660620(0.641/-50.8),rs4041268(0.641/-49),rs11660649(0.641/-41.7),rs4299216(0.641/-51.9),rs11664547(0.641/-50),rs140719534(0.641/-23.1),rs72924158(0.641/-	51450245	51670445	220.2	rs72927946(0.99	51455745	51550045	94.3	-	-	0
rs12713570	2	66875719	0.000001282	-0.034105	0.007	G/A	0.433	0.997	27,651	++++++	rs12713571(0.993/6.22),rs10490190(0.99/27.2),rs1974691(0.988/21.2),rs6720493(0.98/-31.9),rs12713569(0.873/-41.5),rs11695019(0.539/2.77),rs1558718(0.537/21.5),rs1303866(0.531/13.2),rs13006322(0.531/-5.85),rs55907104(0.528/4.65),rs34441555(0.528/28.1),rs13025385(0.528/29.9),rs17628190(0.528/27.9),rs6761229(0.521/-36.1),rs35751375(0.521/-15.7),rs2861111(0.519/-56.7),rs7602403(0.356/-43.1),rs11675077(0.356/27),rs57809212(0.338/-47),rs6754286(0.335/-50.3),rs61448585(0.331/-52.4),rs62146787(0.329/-46.1),rs6546236(0.32/-53.3),rs67054138(0.225/-8.67),rs55803117(0.225/-17.1),rs17032337(0.225/-10),rs9941653(0.225/21.5),rs140249857(0.225/-0.14),rs67981426(0.225/6.56),rs79707217(0.225/0.148),rs68044562(0.225/4.11),rs66923107(0.225/7.37),rs55830965(0.225/22.2),rs9941639(0.225/21),rs9309393(0.225/20.5),rs59788715(0.225/-1.25),rs55805041(0.225/3.4),rs75076546(0.224/-24.2),rs112315907(0.224/-27.1),rs67776981(0.224/-24.8),rs111521547(0.224/-15.7),rs7598188(0.224/-21.6),rs17032335(0.224/-11.8),rs6546239(0.224/-25.6),rs7573609(0.223/-26),rs6741487(0.156/-49.8),rs66533267(0.155/15.1),rs68170325(0.155/4.15),rs67294009(0.154/-10.1),rs192506056(0.154/-24.8),rs113982455(0.154/-10.9),rs67710556(0.154/5.75),rs2192962(0.154/-50.5),rs72808818(0.154/-12.9),rs60931071(0.154/7.44),rs113141186(0.154/-11.4),rs139535049(0.154/-18.8),rs181523914(0.153/-27.9),rs116704653(0.153/-27),rs4505509(0.153/-25.2),rs114128092(0.153/-27),rs60395529(0.153/-35.9),rs56311538(0.153/-22.1),rs55655907(0.152/-39.1)	66819019	66905619	86.6	rs12713571(0.99	66834219	66902919	68.7	-	MEIS1(-24.7),LINC01798(0.0)	3
rs72838240	17	60701586	0.000001523	-0.0917974	0.0191	A/G	0.0532	0.874	15,594	+-?-?-	rs74999220(0.614/2.16),rs113512985(0.596/-53.3),rs112487967(0.591/-48.6),rs113427437(0.455/-196),rs112997374(0.116/-148),rs7223515(0.116/-136),rs9898829(0.116/-147),rs9675244(0.116/-136),rs72835233(0.115/-163),rs7406820(0.115/-148),rs1812692(0.115/-139),rs150830685(0.115/-145),rs112781672(0.114/-163),rs733025(0.11/-166)	60505586	60703746	198.16	rs74999220(0.61	60701586	60703746	2.16	-	TLK2(0.0),MRC2(0.0)	2

rs140240461	7	85100327	0.00000226	-0.154901	0.0327	C/T	0.0179	0.954	15,594	++?+?+	rs117644446(0.899/-43),rs118119665(0.758/-56.3),rs117025998(0.747/58.9),rs117830687(0.728/137),rs10228353(0.423/-40.4),rs77217600(0.423/-19.3),rs11983565(0.423/-42.3),rs111935481(0.423/-35.9),rs76225781(0.422/-14.4),rs73389571(0.422/-13.9),rs74297487(0.421/-33.7),rs17159936(0.42/-29.3),rs10215813(0.42/-29.5),rs11979144(0.392/-51.8),rs2195554(0.387/-31.6),rs1546759(0.387/-28.5),rs2498551(0.383/-20.5),rs2463664(0.383/-20.4),rs2463665(0.383/-20.4),rs1403790(0.383/-13.1),rs2435279(0.382/-44.7),rs1608484(0.381/-44.3),rs1114589(0.381/-43.5),rs1589706(0.381/-43.9)	85044027	85237327	193.3	rs117644446(0.8	85044027	85237327	193.3	-	LINC00972(0.0)	1
rs7534613	1	58971420	0.0000028	-0.155497	0.0332	C/T	0.0176	0.919	15,594	-+?+?+	-	58971420	58971420	0	-	58971420	58971420	0	-	OMA1(0.0)	1
rs62467945	7	114508802	0.000003526	0.0416984	0.009	T/A	0.223	0.896	22,433	--?---	rs6945846(0.805/-106),rs62467929(0.686/-68.1),rs1840660(0.364/-156),rs62467874(0.354/-381),rs2045293(0.319/-159),rs13312596(0.319/-157),rs10500039(0.319/-158),rs13246064(0.312/-161),rs13222712(0.312/-160),rs776472(0.285/-156),rs12705984(0.266/-171),rs17583919(0.238/70.6),rs12705975(0.23/-193),rs1005959(0.23/-206),rs12705974(0.23/-193),rs12705971(0.23/-199),rs12705982(0.23/-173),rs1005958(0.23/-206),rs12705973(0.229/-196),rs13226763(0.229/-187),rs12705981(0.229/-174),rs1378771(0.229/-204),rs60234044(0.229/-206),rs1456021(0.229/-181),rs12671330(0.229/-175),rs12705977(0.229/-177),rs4730637(0.229/-205),rs12705978(0.229/-174),rs4236599(0.228/-191),rs2396766(0.228/-191),rs12154339(0.227/-194),rs2396765(0.225/-204),rs10250103(0.222/-214),rs1563408(0.215/-206),rs12705979(0.215/-174),rs17291908(0.215/-187),rs60603579(0.117/-473)	114035802	114579402	543.6	rs6945846(0.805	114402802	114508802	106	-	-	0
rs77954641	19	33789608	0.000003693	-0.0475949	0.0103	T/G	0.134	0.98	27,651	------	rs111330623(0.998/0.071),rs73024223(0.996/0.586),rs41476047(0.995/1.03),rs12691(0.993/1.52),rs2081045(0.993/-0.598),rs1049969(0.991/2.2),rs34529039(0.991/3.02),rs707656(0.991/1.92),rs8102513(0.99/-2.67),rs8101436(0.99/-2.57),rs34508287(0.989/4.88),rs16967952(0.989/5.63),rs56160221(0.987/-3.4),rs56287732(0.966/10.7),rs73024299(0.956/12.7),rs8103337(0.953/15.2),rs59048871(0.953/13.8),rs8100151(0.953/14.8),rs111859293(0.95/16.7),rs57971673(0.516/31.1),rs74649479(0.319/-2.86),rs41504144(0.317/6.8),rs75061909(0.317/7.09),rs115349677(0.317/7.56),rs79347999(0.316/11.2),rs117035602(0.316/8.87),rs9646647(0.31/16.4),rs75279684(0.31/13.9),rs139037735(0.31/15.6),rs75710158(0.31/13.9),rs60572033(0.308/21.5),rs115178743(0.308/19.8),rs117674966(0.308/19.5),rs116956259(0.308/21),rs78079567(0.239/23.4),rs74954553(0.239/23.3),rs76778925(0.239/24.9),rs75306861(0.238/27.8),rs77949641(0.238/26.7),rs77886265(0.238/30.7),rs116916534(0.238/27),rs145852769(0.238/29.3),rs117915898(0.238/29.4),rs79955346(0.238/27.6),rs75225891(0.238/26.5),rs76243478(0.233/31.9),rs142997057(0.233/32.9),rs137915523(0.233/34.2),rs151115814(0.233/32.9),rs140470768(0.233/32.2),rs117866287(0.233/35.1),rs140086796(0.233/32),rs10451465(0.232/22.2),rs143406390(0.232/34.3),rs74334153(0.23/31.2)	33786208	33824708	38.5	rs111330623(0.9	33786208	33806308	20.1	-	CEBPA(0.0),CEBPA-DT(0.0)	2
rs77736718	3	26882546	0.000005198	-0.112396	0.0247	T/C	0.0319	0.945	15,594	--?-?-	rs12632297(0.583/20.5),rs73063372(0.139/-30.8),rs17288364(0.108/-121)	26761546	26903046	141.5	-	26882546	26882546	0	-	-	0
rs144539704	9	136235935	0.00000524	-0.139699	0.0307	C/T	0.0206	0.934	15,594	++?+?+	rs78711238(0.945/-27.5),rs117308290(0.484/-106),rs117509822(0.472/4.97),rs11791989(0.215/-57.6),rs13301309(0.209/-71.1),rs11793580(0.199/-67),rs7020432(0.198/-70.2)	136129935	136240905	110.97	rs78711238(0.94	136208435	136235935	27.5	-	SURF6(0.0),MED22(0.0),RPL7	13
rs226114	11	110664239	0.000005287	0.0318959	0.007	C/T	0.499	0.966	27,651	------	rs226115(0.978/-0.15),rs226112(0.786/0.691),rs670586(0.549/4.42),rs226164(0.543/22.5),rs226150(0.526/26.2),rs6589176(0.365/-12.8)	110651439	110690439	39	rs226115(0.978/-	110664089	110664930	0.841	-	-	0
rs9976960	21	24618326	0.000005572	-0.0349953	0.0077	T/G	0.288	0.986	27,651	------	rs9979890(0.996/1.5),rs9980697(0.996/1.47),rs9976409(0.841/-9.05),rs1380323(0.839/3.96),rs2061457(0.838/-11.3),rs9305168(0.701/-46.7),rs9981639(0.701/-46),rs7280897(0.7/-47.5),rs2244982(0.7/-18.5),rs985149(0.7/-47.7),rs2827952(0.699/-26.4),rs2827958(0.699/-25),rs2827951(0.699/-26.5),rs1506011(0.698/-50),rs6516558(0.698/-30),rs9305171(0.697/-44.9),rs1153197(0.697/-39.7),rs1506010(0.697/-49.9),rs1573359(0.697/-30.4),rs922081(0.697/-38.5),rs1506012(0.697/-50.1),rs1506009(0.697/-49.8),rs4392338(0.697/-43.4),rs1153203(0.697/-34.7),rs2827950(0.696/-50.2),rs1870464(0.694/-45.4),rs1870463(0.694/-45.4),rs9305166(0.694/-47.2),rs9982946(0.694/-45.4),rs9305167(0.693/-47.1),rs10854271(0.67/-27.4),rs9680422(0.67/-28.1),rs1153199(0.662/-38.7),rs4816894(0.657/-7.6),rs1870470(0.656/-7.43),rs8127651(0.655/-54.1),rs7276428(0.65/-69.9),rs2827948(0.649/-50.3),rs2827983(0.638/-9.63),rs2827979(0.637/-15.8),rs2827965(0.637/-22),rs13046121(0.635/-14.3),rs1304747(0.635/-33.1),rs2408668(0.635/-42.9),rs2169040(0.594/-2.28),rs198053(0.591/-105),rs2827987(0.579/0.523),rs1903161(0.561/7.25),rs2827992(0.555/5.56),rs4816896(0.543/9.15),rs2827995(0.543/8.99),rs2827994(0.542/8.85),rs768176(0.537/9.44),rs1380324(0.536/9.79),rs2226715(0.423/10.7),rs1380322(0.364/3.37),rs8133976(0.363/0.058),rs985211(0.363/1.91),rs1459399(0.362/-5.15),rs8132371(0.362/-1.57),rs1459402(0.362/-9.11),rs9977321(0.361/-14.1),rs2408670(0.361/-13),rs7282152(0.361/-8.95),rs1459400(0.361/-5.24)	24513326	24629026	115.7	rs9979890(0.996	24548426	24622286	73.86	-	-	0
rs4817234	21	15899085	0.00000666	-0.0366951	0.0082	A/G	0.248	0.942	27,651	------	rs9979080(0.943/3.2),rs4817235(0.888/0.138)	15899085	15902285	3.2	rs9979080(0.943	15899085	15902285	3.2	-	SAMSN1(0.0)	1
rs12708476	15	63293959	0.000007488	0.0314018	0.007	G/A	0.468	0.98	27,651	------	rs12911467(0.684/-0.778),rs12903994(0.683/-2.08),rs34642222(0.655/-3.38),rs12913459(0.617/-3.59),rs11852619(0.304/-8.12),rs62011025(0.297/-17),rs17751112(0.294/-23.6),rs13380382(0.272/-8.38),rs17823147(0.266/-40),rs4352001(0.259/-32.3),rs28707628(0.258/-46.6),rs4775571(0.258/-43.6),rs2414805(0.257/-46.5),rs12900263(0.256/-46.7),rs35317361(0.255/-43.6),rs36030007(0.254/-26.8),rs2130162(0.253/-31.1),rs17750687(0.253/-28.9),rs12708475(0.253/-28.8),rs11631647(0.145/-37.1),rs28742917(0.123/21.5)	63247259	63315459	68.2	rs12911467(0.68	63290369	63293959	3.59	-	TPM1(0.0),TPM1-AS(0.0)	2

rs148351785	6	134127406	0.000007711	-0.155205	0.0347	A/C	0.0155	0.95	17,369	---??-	rs146099425(0.998/15.7),rs142898776(0.998/6.99),rs140466384(0.998/6.94),rs116851966(0.996/8.72),rs139120436(0.996/-0.923),rs138381522(0.996/-3.51),rs143307852(0.99/-11.8),rs143142433(0.986/-20.3),rs148236974(0.984/-20),rs117728942(0.977/-12),rs143798372(0.973/-28.4)	134099006	134143106	44.1	rs146099425(0.9	134099006	134143106	44.1	-	TARID(0.0),LINC01312(0.0)	2
rs75688327	15	47351247	0.000007769	-0.0488962	0.0109	A/G	0.137	0.88	22,433	--?---	rs1912977(0.317/-92.1),rs72729727(0.317/-99.7),rs4775660(0.317/-90.5),rs112835629(0.317/-81.4),rs2175575(0.317/-85.1),rs72729715(0.313/-113),rs72729717(0.313/-110),rs72729718(0.313/-109),rs10519093(0.313/-110),rs17279867(0.313/-112),rs55957745(0.313/-103),rs72729726(0.312/-101),rs16958906(0.312/-116),rs35378533(0.311/-102),rs4774487(0.311/-116),rs968233(0.311/-101),rs17279366(0.309/-124),rs16958894(0.309/-124),rs72727802(0.308/-127),rs72729720(0.308/-107),rs58025235(0.308/-131),rs17345891(0.308/-103),rs72727794(0.308/-133),rs72729722(0.308/-106),rs17345773(0.308/-109),rs1355589(0.308/-105),rs72729706(0.308/-124),rs17280083(0.308/-106),rs60580630(0.308/-105),rs11854171(0.306/-68.3),rs11857329(0.306/-68.3),rs4775663(0.306/-66.5),rs55927419(0.305/-64.7),rs72729723(0.292/-104),rs72729724(0.291/-104),rs72729725(0.29/-104),rs72727775(0.288/-148),rs56300189(0.288/-147),rs11855029(0.288/-164),rs73390803(0.287/-149),rs12148286(0.287/-151),rs56166124(0.287/-148),rs12148629(0.287/-142),rs11070560(0.255/-94.3),rs1918662(0.189/-257),rs17321993(0.175/-321),rs17270239(0.151/-268),rs10519079(0.15/-273),rs17268753(0.15/-326),rs2413831(0.15/-246),rs2413828(0.15/-309),rs2413832(0.15/-245),rs12902918(0.15/-270),rs66691168(0.15/-303),rs67761126(0.149/-320),rs12902816(0.149/-284),rs11854431(0.145/-265),rs12902436(0.145/-270),rs12912198(0.145/-247),rs4517728(0.145/-270)	47025247	47351247	326	-	47351247	47351247	0	-	-	0
rs12410615	1	57231991	0.00000788	-0.0380031	0.0085	A/G	0.218	0.988	27,651	------	rs1342381(0.997/-9.61),rs12022804(0.997/-9.03),rs2298127(0.996/-10.1),rs12410059(0.996/-6.29),rs12405047(0.785/-6.28),rs1578677(0.785/-4.06)	57221891	57231991	10.1	rs1342381(0.997	57221891	57231991	10.1	-	PRKAA2(-1.0),FYB2(0.0)	2
rs79786919	2	47994675	0.000008542	0.115398	0.0259	T/C	0.029	0.9	15,594	-+?+?+	rs114144926(0.663/17),rs78731390(0.656/13.6),rs75733466(0.17/358),rs12053194(0.133/342)	47994675	48352675	358	rs114144926(0.6	47994675	48011675	17	-	MSH6(0.0),FBXO11(0.0)	2
rs79269825	16	62453410	0.000009247	-0.138504	0.0312	T/C	0.0205	0.928	14,988	?-?-?-	rs16964607(0.825/-36.9),rs117935849(0.795/-24.2),rs7194212(0.494/-60.5),rs6498848(0.494/-60.6),rs7194729(0.494/-60.3),rs16964587(0.493/-62.9),rs16964590(0.492/-62.8)	62390510	62453410	62.9	rs16964607(0.82	62416510	62453410	36.9	-	-	0
rs76612314	4	10209522	0.000009445	0.0733991	0.0166	A/G	0.0725	0.859	15,594	++?+?+	rs76835013(0.884/151),rs150196557(0.514/61.2),rs141474550(0.307/149),rs149844708(0.201/-152)	10057522	10360522	303	rs76835013(0.88	10209522	10360522	151	-	-	0
9:25926170_C_(	9	25926170	0.000009717	0.100198	0.0226	G/C	0.0386	0.966	15,594	--?-?-	rs10491877(0.975/-13.3),rs10812323(0.966/-10.7),rs7856714(0.964/-8.26),rs10967112(0.961/-26.1),rs10812324(0.96/7.26),rs7028344(0.956/5.2),rs75159073(0.955/2.87),rs77022124(0.953/-4.92),rs7026829(0.949/-12.9),rs7038828(0.939/-32.4),rs150384310(0.561/-5.68),rs1219936(0.281/-24.5),rs666778(0.264/-2.91),rs1219932(0.234/-19),rs644506(0.221/1.03),rs702228(0.221/-3.33),rs504517(0.221/-2.44),rs1782572(0.22/-11.4),rs542788(0.219/2.33),rs1219931(0.217/-17.5),rs562342(0.217/-0.669),rs658858(0.216/2.98),rs836957(0.216/-26.3),rs861359(0.214/1.32),rs636294(0.207/-3.76)	25893770	25933430	39.66	rs10491877(0.97	25893770	25933430	39.66	-	-	0

Supplementary Table S3: Results from the PRS analysis for HS (Leave-one-out), obsessive-compulsive disorder (OCD), depressive disorder (DEP), schizophrenia (SCZ), autism-spectrum disorder (ASD), attention-deficit hyperactivity disorder (ADHD), and educational attainment (EA). Results are presented for every target dataset (STR (combination of the three STR datasets), NTR, TwinsUK, and SfS), across pre-selected p-value thresholds (P Threshold). Listed are the Beta, standard error (SE), Z-score (Z), and p-value (P) from the regression. For STR, NTR, and TwinsUK the effective N was determined based on the actual N (including family members) weighted by the ratio of the squared SEs from the GEE sandwich-corrected model and the naive model (no correction). For SfS the sample N is listed. Bonferroni-corrected significant p-values (<0.05/7=0.00714) are in bold.

Discovery	Target	P Threshold	Neffective (target)	Beta	SE	Z	P
Hoarding Symptoms (Leave-one-out)							
**HS**	**STR**	0.5	11052.18	0.00181	0.00705	0.25689	0.79726
	**NTR**		5759.06	-0.00096	0.01101	-0.08741	0.93035
	**TwinsUK**		3359.35	0.00618	0.01288	0.48005	0.63119
	**SfS**		5218	0.00993	0.01374	0.72246	0.47005
Cross-trait							
**OCD**	**STR**	0.1	11131.88	-0.00439	0.00714	-0.61446	0.53891
	**NTR**		5717.05	0.00677	0.01128	0.60057	0.54813
	**TwinsUK**		3398.42	0.01034	0.01279	0.80845	0.41883
	**SfS**		5218	0.00993	0.01374	0.72246	0.47005
**DEP**	**STR**	0.05	10720.69	0.02448	0.00718	3.4082	**0.00065**
	**NTR**		6118.68	0.00915	0.01112	0.82286	0.41059
	**TwinsUK**		3548.09	-0.03323	0.01253	-2.65233	**0.00799**
	**SfS**		5218	0.027333	0.01378	1.9842	**0.04729**
**SCZ**	**STR**	0.1	10680.06	0.03533	0.00747	4.72848	**1x10-06**
	**NTR**		6071.04	0.01336	0.01198	1.11494	0.26488
	**TwinsUK**		3578.2	0.02807	0.01399	2.00715	**0.04473**
	**SfS**		5218	0.0025	0.0141	0.17727	0.85931
**ASD**	**STR**	0.1	11332.74	0.01465	0.00697	2.10171	**0.03558**
	**NTR**		5981.45	-0.0023	0.01126	-0.2046	0.83789
	**TwinsUK**		3444.56	0.0202	0.01295	1.56053	0.11863
	**SfS**		5218	0.0328	0.01416	2.31679	**0.02055**
**ADHD**	**STR**	0.1	11173.34	0.01874	0.00706	2.65613	**0.0079**
	**NTR**		6098.19	-0.00152	0.01111	-0.13706	0.89098
	**TwinsUK**		3448.24	-0.00913	0.01288	-0.70911	0.47826
	**SfS**		5218	-0.01176	0.01384	-0.84986	0.39544
**EA**	**STR**	1	10962.42	0.00749	0.00707	1.05896	0.28962
	**NTR**		5786.57	0.02905	0.01121	2.59051	**0.00958**
	**TwinsUK**		3340.07	0.05878	0.01298	4.5304	**6x10-06**
	**SfS**		5218	0.02837	0.01472	1.92701	0.05403

Supplementary Table S4: Results of sign-test analysis for STR, NTR, SfS, and TwinsUK for three p-value thresholds (P_TH) 1e-06, 1e-05, and 1e-04. In the column "Discovery" are the discovery cohorts, in the coliumns "Replication" the target datasets. Nsum denotes the number of genomic regions in the replication study at each p-value threshold for which results are present. Npos is the number of genomic regions (in the replication study) that have the same direction with respect to the discovery results. The ratio is npos/nsum and sign-test is the P-value associated with the sign test. A ratio above 0.5 indicates a positive signtest, while a ratio below 0.5 indicates more divergence in the signtest than convergence.

		**Replication**															
		**STR**				**NTR**				**SfS**				**TwinsUK**			
**Discovery**	**P_TH**	**npos**	**nsum**	**sign-test**	**ratio**	**npos**	**nsum**	**sign-test**	**ratio**	**npos**	**nsum**	**sign-test**	**ratio**	**npos**	**nsum**	**sign-test**	**ratio**
**STR**	**0.0001**					54	123	0.92559188	0.44	53	109	0.64909441	0.49	90	183	0.61621868	0.49
**STR**	**0.00001**					7	20	0.94234085	0.35	10	18	0.40726471	0.56	12	29	0.86753455	0.41
**STR**	**0.000001**					1	2	0.75	0.5	1	2	0.75	0.5	0	3	1	0
**NTR**	**0.0001**	54	123	0.92559188	0.44					27	53	0.5	0.51	39	69	0.16777881	0.57
**NTR**	**0.00001**	7	20	0.94234085	0.35					1	5	0.96875	0.2	4	5	0.1875	0.8
**NTR**	**0.000001**	1	2	0.75	0.5					0	1	1	0	0	1	1	0
**SfS**	**0.0001**	31	76	0.957677	0.41	30	67	0.83578397	0.45					33	76	0.89663158	0.43
**SfS**	**0.00001**	4	12	0.92700195	0.33	6	12	0.61279297	0.5					5	12	0.80615234	0.42
**SfS**	**0.000001**	0	0	1	NA	0	0	1	NA					0	0	1	NA
**TwinsUK**	**0.0001**	68	138	0.60072773	0.49	46	85	0.25771287	0.54	42	80	0.36877715	0.53				
**TwinsUK**	**0.00001**	8	17	0.68547058	0.47	6	10	0.37695313	0.6	5	11	0.72558594	0.45				
**TwinsUK**	**0.000001**	1	2	0.75	0.5	1	2	0.75	0.5	0	1	1	0				

Supplementary Table S5: Results of sign-test analyses for STR. In the upper half, the discovery datasets are STR-only leave one out (LOO) analyses, in the lower half of the table, the discovery datasets are meta-analyses of NTR, TwinsUK, and LOO STR. Target/replication datasets are the individual STR datasets separated by cohort/age groups (Catss15 mean age = 15.47 (SD = 0.36); Catss18 mean age= 18.56 (SD = 0.33); Catss24 mean age = 23.84 (SD = 0.32); Yatss mean age = 23.93 (SD = 1.78) to determine if there was any pronounced age-related effect in the STR data. Sign-tests were performed for three different p-value thresholds (P_TH) 1e-06, 1e-05, and 1e-04. Nsum denotes the number of genomic regions in the replication study at each p-value threshold for which results are present. Npos is the number of genomic regions (in the replication study) that have the same direction with respect to the discovery results. The ratio is npos/nsum and 'sign-test' the P-value associated with the sign test. A ratio above 0.5 indicates a positive signtest, while a ratio below 0.5 indicates more divergence in the signtest then convergence.

Base	Target	P_TH	npos	nsum	sign-test	ratio
**STR only**						
Catss18PC_Catss24PC_Yatss	STR_Catss15_PC	0.0001	101	194	0.30769037	0.52
Catss18PC_Catss24PC_Yatss	STR_Catss15_PC	0.00001	11	19	0.32380295	0.58
Catss18PC_Catss24PC_Yatss	STR_Catss15_PC	0.000001	2	2	0.25	1
Catss15PC_Catss24PC_Yatss	STR_Catss18_PC	0.0001	99	191	0.33214799	0.52
Catss15PC_Catss24PC_Yatss	STR_Catss18_PC	0.00001	9	23	0.89498019	0.39
Catss15PC_Catss24PC_Yatss	STR_Catss18_PC	0.000001	0	5	1	0
Catss15PC_Catss18PC_Yatss	STR_Catss24_PC	0.0001	86	179	0.72500228	0.48
Catss15PC_Catss18PC_Yatss	STR_Catss24_PC	0.00001	13	22	0.26173353	0.59
Catss15PC_Catss18PC_Yatss	STR_Catss24_PC	0.000001	3	6	0.65625	0.5
Catss15PC_Catss18PC_Catss24PC	STR_Yatss	0.0001	100	190	0.25695482	0.53
Catss15PC_Catss18PC_Catss24PC	STR_Yatss	0.00001	18	29	0.13246545	0.62
Catss15PC_Catss18PC_Catss24PC	STR_Yatss	0.000001	0	3	1	0
**All cohorts (replicating separate STR)**						
NTR_TwinsUK_STRCatss18PC_Catss24PC_Yatss	STR_Catss15PC	0.0001	85	155	0.13036604	0.55
NTR_TwinsUK_STRCatss18PC_Catss24PC_Yatss	STR_Catss15PC	0.00001	8	13	0.29052734	0.62
NTR_TwinsUK_STRCatss18PC_Catss24PC_Yatss	STR_Catss15PC	0.000001	2	3	0.5	0.67
NTR_TwinsUK_STRCatss15PC_Catss24PC_Yatss	STR_Catss18PC	0.0001	86	176	0.64681458	0.49
NTR_TwinsUK_STRCatss15PC_Catss24PC_Yatss	STR_Catss18PC	0.00001	8	17	0.68547058	0.47
NTR_TwinsUK_STRCatss15PC_Catss24PC_Yatss	STR_Catss18PC	0.000001	0	1	1	0
NTR_TwinsUK_STRCatss15PC_Catss18PC_Yatss	STR_Catss24PC	0.0001	86	168	0.40852324	0.51
NTR_TwinsUK_STRCatss15PC_Catss18PC_Yatss	STR_Catss24PC	0.00001	12	20	0.25172234	0.6
NTR_TwinsUK_STRCatss15PC_Catss18PC_Yatss	STR_Catss24PC	0.000001	2	3	0.5	0.67
NTR_TwinsUK_STRCatss15PC_Catss18PC_Catss24PC	STR_Yatss	0.0001	84	154	0.14740676	0.55
NTR_TwinsUK_STRCatss15PC_Catss18PC_Catss24PC	STR_Yatss	0.00001	6	12	0.61279297	0.5
NTR_TwinsUK_STRCatss15PC_Catss18PC_Catss24PC	STR_Yatss	0.000001	3	4	0.3125	0.75

Supplementary Table S6: Results of sign-test analyses for NTR, separated into three age groups (NTR < 30 years, NTR 30-45 years, NTR > 45 years). The discovery datasets are the over-all STR dataset, as well as the STR sub-cohorts separated by age. This was done to determine if there were any pronounced age-related effect in the NTR data. Sign-tests were performed for four different p-value thresholds (P_TH) 5e-08, 1e-06, 1e-05, and 1e-04. Nsum denotes the number of genomic regions in the replication study at each p-value threshold for which results are present. Npos is the number of genomic regions (in the replication study) that have the same direction with respect to the discovery results. The ratio is npos/nsum and sign-test the P-value associated with the sign test. A ratio above 0.5 indicates a positive signtest, while a ratio below 0.5 indicates more divergence in the signtest then convergence.

		NTR < 30				NTR 30-45				NTR > 45			
**Discovery**	**P_TH**	**npos**	**nsum**	**sign-test**	**ratio**	**npos**	**nsum**	**sign-test**	**ratio**	**npos**	**nsum**	**sign-test**	**ratio**
**TwinsUK**	**0.0001**	42	83	0.5	0.51	50	85	0.0641982	0.59	44	86	0.45710582	0.51
**TwinsUK**	**0.00001**	6	10	0.37695313	0.6	6	10	0.37695313	0.6	6	11	0.5	0.55
**TwinsUK**	**0.000001**	1	2	0.75	0.5	1	2	0.75	0.5	1	2	0.75	0.5
**STR**	**0.0001**	61	125	0.63966374	0.49	57	123	0.81635163	0.46	55	123	0.89667645	0.45
**STR**	**0.00001**	11	20	0.41190147	0.55	10	20	0.58809853	0.5	8	20	0.86841202	0.4
**STR**	**0.000001**	2	2	0.25	1	0	2	1	0	1	2	0.75	0.5
**Catss15**	**0.0001**	46	88	0.3746645	0.52	50	89	0.14454804	0.56	37	89	0.9553396	0.42
**Catss15**	**0.00001**	3	9	0.91015625	0.33	6	9	0.25390625	0.67	3	9	0.91015625	0.33
**Catss15**	**0.000001**	0	0	1	NA	0	0	1	NA	0	0	1	NA
**Catss18**	**0.0001**	47	94	0.54103847	0.5	47	91	0.41704052	0.52	50	91	0.2009064	0.55
**Catss18**	**0.00001**	5	9	0.5	0.56	3	8	0.85546875	0.38	1	9	0.9980469	0.11
**Catss18**	**0.000001**	0	1	1	0	0	1	1	0	1	1	0.5	1
**Catss24**	**0.0001**	45	87	0.4151865	0.52	36	84	0.92217177	0.43	39	84	0.77739767	0.46
**Catss24**	**0.00001**	4	10	0.828125	0.4	4	10	0.828125	0.4	2	10	0.9892578	0.2
**Catss24**	**0.000001**	0	0	1	NA	0	0	1	NA	0	0	1	NA
**Yatss**	**0.0001**	54	130	0.9783724	0.42	65	130	0.53492233	0.5	71	129	0.14534393	0.55
**Yatss**	**0.00001**	11	18	0.24034119	0.61	10	18	0.40726471	0.56	11	18	0.24034119	0.61
**Yatss**	**0.000001**	2	2	0.25	1	1	2	0.75	0.5	1	2	0.75	0.5
